# An insight into the invasion of breast ductal carcinoma *in situ* based on clinical, pathological and hematological data

**DOI:** 10.7717/peerj.13966

**Published:** 2022-08-31

**Authors:** Yanbiao Liu, Zining Jin, Xinmiao Yu, Ang Zheng, Feng Jin, Xu Wang

**Affiliations:** Department of Breast Surgery, The First Hospital of China Medical University, Shenyang, Liaoning, China

**Keywords:** Ductal Carcinoma *in situ*, Breast cancer, Invasion, Pathological marker, Hematological indicator

## Abstract

**Background:**

Ductal carcinoma *in situ* (DCIS) has become a non-negligible part of breast cancers owing to the greatly increased incidence. While its natural history was not fully elucidated, which is the reason for current controversies in clinical treatment. Exploration of this issue from a clinical perspective is meaningful.

**Methods:**

Medical records of 389 patients diagnosed with DCIS or DCIS with invasive ductal carcinoma (IDC) were reviewed. All of them received appropriate medical care in our center. All 324 patients in training cohort were divided into invasion and non-invasion groups based on pathology. Differences in DCIS immunohistochemical markers and hematological indicators between them were analyzed. In the invasion group, differences between DCIS and matched IDC were compared to explore changes in the tumor heterogeneity during invasion. Conclusions are validated in the validation cohort of 65 patients.

**Results:**

Patients in invasion and non-invasion groups were balanced in baseline characteristics and no statistically significant differences were noticed for DCIS immunohistochemical markers. For hematological indicators, high expression of platelet >291.50) (odds ratio, 2.46; CI [1.35–4.46]; *p* = 0.003) and SII (>347.20) (odds ratio, 2.54; CI [1.56–4.12]; *p* < 0.001) were established as independent predictors for invasion by logistic analysis and were validated in the validation cohort. Ki-67 of IDC was significantly higher than that of matched DCIS (*p* < 0.001). HER2 expression and histological grade of DCIS were separately linearly related to those of IDC.

**Conclusion:**

The change in hematological indicators is an independent predictor for invasion and can be incorporated into the treatment decision-making process for DCIS. Invasion tumor cells exhibit a stronger proliferative capacity compared with the *in-situ* ones. There are linear relationships in HER2 expression and histological grades between DCIS and matched IDC. DCIS subclones with different histological grades will develop into invasive carcinomas separately.

## Introduction

Ductal carcinoma *in situ* (DCIS), a once-rare disease, has increased in incidence over the past few decades due to the dramatic advance in mammography (Swallow et al., 1996; Oseni et al., 2019). Today, accounting for about one-fifth of new cases each year, DCIS has become a non-negligible part of breast cancers (Sung et al., 2021). Studies have revealed that patients with unrecognized DCIS who are excised as benign lesions progress to invasive ductal carcinoma (IDC) in 39–60% of cases, which supported the hypothesis that DCIS was a precursor lesion for most invasive ductal carcinomas (Collins et al., 2005; Sanders et al., 2005).

Endeavors have been made to improve our knowledge of DCIS. By analyzing 300 patients with synchronous DCIS and IDC, Gupta et al. (1997) concluded that IDCs with different features were developed from corresponding DCIS subclones separately, with biological and genetic features already established in the pre-invasion stage. Latta et al. (2002) reviewed medical records of 251 patients and noted that overexpression of HER2 was much higher in DCIS than in the synchronous IDC. They further concluded that amplification of HER2 enhanced survival and reproduction capabilities of DCIS cells in an ischemic environment, but was not involved in the invasion process. Yu et al. (2011) compared the distribution differences of breast cancer immunophenotyping among DCIS, DCIS with microinvasion and DCIS with invasion component and proposed a scenario that HER2-overexpressed and ER-negative tumor cells possessed enhanced invasion capacity. Considerable on-going studies have deciphered the ability of tumor cells inside mammary ducts to respond to extracellular matrix stimuli and reactively adjust energy metabolism and cytoskeletal architecture to enhance their invasion and migration capabilities (Zanotelli, Zhang & Reinhart-King, 2021).

However, the current knowledge is only a tip of the iceberg with uncertainty. Like IDC, DCIS is a collection of different subclones with high heterogeneity. It is speculated that only about half of the DCIS subclones are aggressive, while the exact one remains unrevealed (Barrio & Van Zee, 2017). Endeavors have been made to identify biomarkers that can be used to stratify DCIS for invasion and recurrence risk but have not yet been successful.

Ignorance of the biological behavior of tumor cells led to controversies over clinical treatment options.

Mastectomy has been considered curative, yet it appears to be immoderate because DCIS is a confined disease surrounded by the basement membrane. However, compared with mastectomy, for patients undergoing lumpectomy alone, in-breast tumor recurrence (IBTR) occurred more frequently even though complete resection was guaranteed (Barrio & Van Zee, 2017). Clinical trials have demonstrated that the addition of radiotherapy or endocrine therapy after breast-conserving surgery (BCS) decreased both ipsilateral and contralateral IBTR for some patients, which, although positive for the treatment, has not been fully elucidated by specific mechanisms (Fisher et al., 1993; Fisher et al., 1999; Houghton, 2003).

Continuous observation of DCIS cell invasion is impossible because patients diagnosed with DCIS are recommended to undergo appropriate surgery in the current medical setting (Hong et al., 2018). As for those with recurrent IDC after DCIS surgery, suspicions retained. Firstly, it is a mystery whether the recurrent IDC develops from the residual DCIS component or is just a second primary carcinoma. Second, there is often a long period between the initial diagnosis of DCIS and the relapse of IDC. Visser et al. (2019) followed 155 patients with DCIS who underwent lumpectomy alone, and the meantime to recurrence of the invasion carcinoma was 6.3 years. This period is now even longer according to the NCCN guideline recommendations for the treatment of DCIS (mastectomy or lumpectomy followed by radiation treatment, with or without sentinel lymph node biopsy) (Gradishar et al., 2020). Over such a long period, we are unable to assess the impact of external factors (like disease conditions, drug effects, *etc*.) and natural changes in body function on the subject. Therefore, we consider those with synchronous DCIS and IDC components to be more suitable for this study.

In this study, we analyzed electronic medical records of 389 patients diagnosed with DCIS or DCIS with IDC; compared differences in clinical, pathological, and hematological data between them; and explored changes in tumor heterogeneity during invasion, aiming to investigate which DCIS subclones exhibit a greater invasion ability; what changes in tumor heterogeneity are brought about and what role does the immune and inflammatory system play in the invasion process.

## Materials & Methods

### Inclusion and exclusion criteria

We adopted strict inclusion and exclusion criteria to ensure the rigor of this study. All enrolled patients met the following criteria: (a) Female gender with unilateral breast cancer; (b) initial diagnosis of breast cancer without evidence of distant metastasis; (c) pure DCIS or synchronous DCIS and IDC (referring to invasive carcinoma or microinvasive carcinoma) in breast lesions confirmed by puncture biopsy or excisional biopsy.

Patients with the following characteristics were excluded; (a) Concomitant lobular carcinoma; (b) concomitant invasive carcinoma in any other lesions and those who had already received systemic therapy; (c) failure to do immunohistochemical staining or those with pathology reports which did not indicate whether the immunohistochemistry was part of the DCIS component or the IDC component; (d) had severe blood disorders.

### Enrolled patients

**Figure 1:**
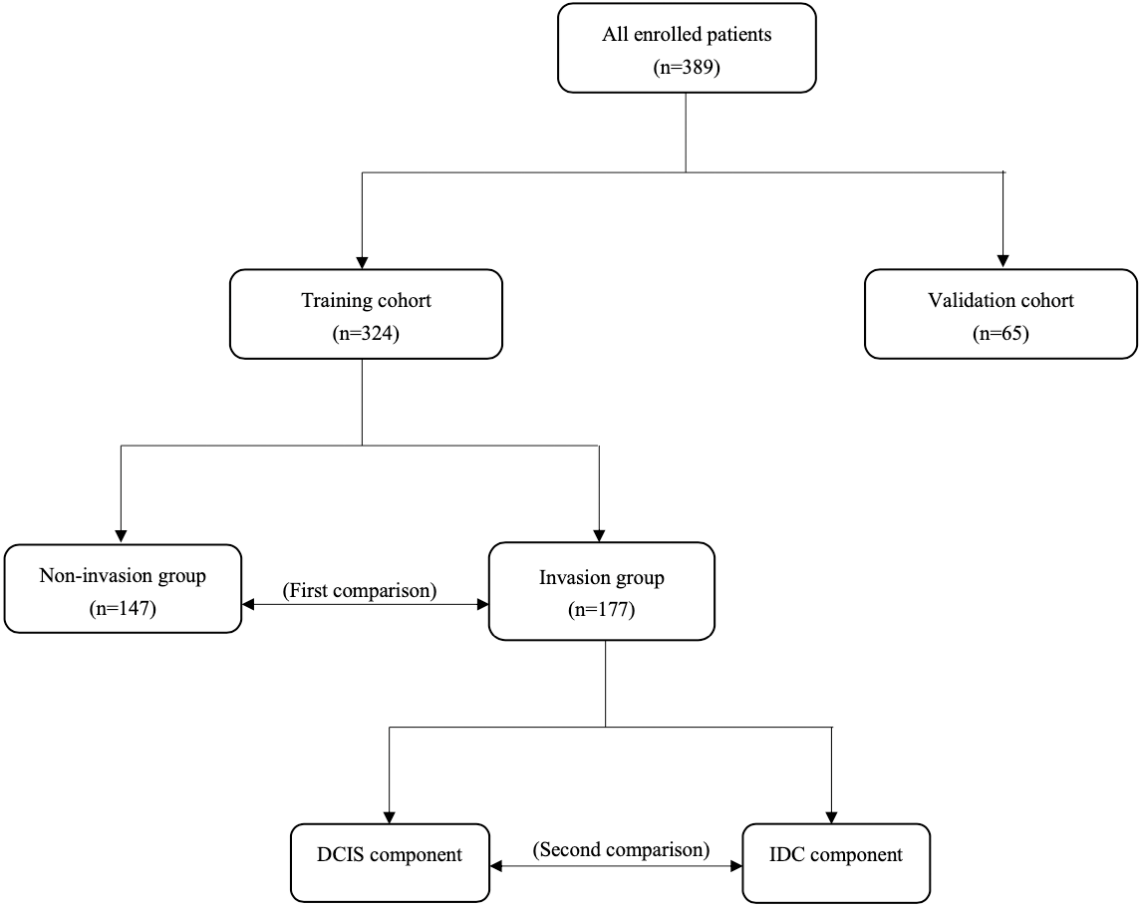
Study design.

From 2012 to 2020, a total of 324 patients hospitalized in the Department of Breast Surgery at the First Hospital of China Medical University were enrolled to form the training cohort. From 2020 to 2021, an additional 65 patients were enrolled to form the validation cohort. All of them have received appropriate medical care including mastectomy/lumpectomy combined with sentinel lymph node biopsy (SLNB)/axillary lymph node dissection (ALND). Based on pathology, we first divided patients in the training cohort into invasion and non-invasion groups in order to investigate which subclones of tumor cells are more aggressive and what will occur in the immune and inflammatory system in the invasion process through comparing differences in DCIS immunohistochemical markers and hematological indicators between them. Second, we compared differences in immunohistochemical markers between DCIS and matched IDC components in the invasion group with an aim to explore changes in the heterogeneity of tumor cells during invasion. Conclusions are validated in the validation cohort (Fig. 1). All patients consented to their clinical information being used in this study and have signed a consent form. This study was approved by the Ethics Committee of China Medical University (Approval number: AF-SOP-07−1.1-01).

### Pathological diagnosis

The handling of pathological specimens and immunohistochemical staining were performed as previously described (Liu et al., 2021). At least two experienced pathologists have independently reviewed and signed off on the report, and disputes between them were resolved by negotiation. According to American Society of Clinical Oncology and College of American Pathologists guidelines, expression of ER and PR was mainly based on the percentage of immunohistochemical (IHC)-stained positive nuclei and the intensity of staining with positive of them meaning that ≥ 1% of tumor cell nuclei was immunoreactive (Hammond et al., 2010). Expression of HER2 at the protein level was explored by IHC and was classified into 4 classes (0+, 1+, 2+ and 3+) based on the percentage of positively stained cells, morphology, and staining intensity of the cell membrane (Schaller et al., 2001). Detection of HER2 gene (ERBB2) amplification has also been used by pathologists as an adjunct in combination with IHC to make a more precise determination of HER2 expression (Wesoła & Jeleń, 2015). However, because anti-HER2 therapy has not been shown to be clearly beneficial in DCIS, gene expression testing is not routinely performed (Siziopikou et al., 2013). Therefore, we only discuss its expression on the protein level in this study.

Ki-67 is a biomarker responding to the proliferative activity of tumor cells (Chen & Wu, 2015). In routine pathological examinations, test for Ki-67 was performed and the percentage of positively stained cells was reported. In the present study, 30% was regarded as a cut-off value to distinguish between high and low expression.

Based on the modified Scarff-Bloom-Richardson grading system, pathologists scored specimens on adenoid formation, nuclear morphology, and nuclear schizograms respectively, then classified IDC into three histological categories (Grade 1, 2, and 3) on this basis (Elston & Ellis, 1991; Bansal et al., 2012). Similarly, DCIS was classified into three grades (Low, Medium, and High) based on a grading pattern containing assessment of nuclear grading, necrosis, nuclear splitting images and histology. In the process of data collection, we noted the coexistence of two or more histological grades in some DCIS lesions. We sought to explore its impact on invasion.

### Collection of blood sample

Hematological data were obtained by searching the electronic medical records of patients for the period of their hospitalization. We reviewed the platelet count, lymphocyte count, and neutrophil count in peripheral blood samples and derived three hematological indexes: platelet lymphocyte ratio (PLR), neutrophil-lymphocyte ratio (NLR), and systemic inflammatory index (SII) on this basis. PLR was calculated as platelet count/lymphocyte count. NLR was calculated as neutrophil count/lymphocyte count. SII was calculated as neutrophil count × platelet count/lymphocyte count.

### Statistical analysis

In the first comparison, age was the only continuous variable conforming to a normal distribution, and the student’s *t*-test was used for its statistical analysis. Mann–Whitney U test was used to analyze the statistical differences among the remaining non-normally distributed continuous variables and hierarchical variables. Pearson’s chi-square test was used for statistical analysis among categorical variables. Logistic regression models were used to further specify independent influencing factors. Variables with *p* < 0.1 in univariate analysis were adopted in subsequent multivariate analysis. In the second comparison, Wilcoxon’s test and McNamar’s test were used for statistical analysis of non-normally distributed continuous variables and categorical variables. Kendall’s tau-U rank correlation and linear by linear chi-square test were used to investigate the correlation between HER2 and pathological grade and their co-varying trends respectively. Continuous variables conforming to a normal distribution were presented as mean ±standard deviation (SD), otherwise, they are presented through median joint quartiles. All statistical analyses were two-tailed, and *p* < 0.05 was considered statistically significant. All statistical analyses were performed using SPSS software version 26.0 (IBM SPSS, Inc., Chicago, IL, USA).

## Results

### Differences between invasion and non-invasion groups in baseline characteristics

The mean age of all patients in the training cohort was 51.78 ± 11.40. One hundred and seventy-seven patients were divided into the invasion group and the other 147 patients were divided into the non-invasion group, with a ratio of 1.2:1. Detailed differences between them were shown in Table 1. The mean age of patients was 51.49 ± 10.97 in the invasion group and 52.14 ± 11.92 in the non-invasion group. Although patients in the invasion group were younger than those in the non-invasion group, the difference was negligible without statistical difference (*p* = 0.61). Interestingly, we noted a higher proportion of menopausal patients in the invasion group with a younger mean age compared with the non-invasion group (47.46% vs. 39.46%). This anomaly, although is not statistically significant (*p* = 0.10), does seem to suggest the important role of the endocrine factor in breast cancer. As for the surgical procedure, the proportion of patients in the non-invasion group who received lumpectomy and SLNB were both higher than those in the invasion group, although the differences were not statistically significant (36.05% vs. 29.38% *p* = 0.20 and 40.13% vs. 33.90% *p* = 0.25). In both groups, the proportion of left-sided breast cancers was higher than that of right-sided breast cancers (58.19% vs. 41.81% for the invasion group and 53.06% vs. 46.94% for the non-invasion group) but was not statistically significant (*p* = 0.33). We considered the two groups were balanced in baseline characteristics.

**Table 1:** Characteristics between invasion and non-invasion groups.

Characteristics	Invasion group (*n* = 177)	Non-invasion group (*n* = 147)	*P*-value
Age (*n* = 324)	51.49 ± 10.97	52.14 ± 11.92	0.61
Anatomic subdivisions (*n* = 323)			
Left	103	78	
Right	73	69	0.33
Menstruation (*n* = 320)			
Pre-menopause	89	89	
Menopause	84	58	0.10
Breast Surgery (*n* = 324)			
Mastectomy	125	94	
Lumpectomy	52	53	0.20
Axillary Surgery (*n* = 324)			
ALND	117	88	
SLNB	60	59	0.25
ER of DCIS (*n* = 322)			
Negative	55	44	
Positive	121	102	0.83
PR of DCIS (*n* = 320)			
Negative	50	38	
Positive	124	108	0.59
Her-2 of DCIS (*n* = 322)			
0+	13	14	
1+	28	24	
2+	62	46	
3+	74	61	0.76
Ki-67 of DCIS (*n* = 323)			
High	28	23	
Low	148	124	0.95
DCIS grade (*n* = 271)			
Low	14	26	
Intermediate	29	20	
High	96	86	0.27
Mixture of DCIS grade (*n* = 324)			
Present	8	4	
Absent	169	143	0.39
Platelet (*n* = 321)	255(208,301)	237(206.5,274.5)	0.03
Lymphocyte (*n* = 321)	1.87(1.56,2.34)	1.85(1.55,2.27)	0.67
Neutrophil (*n* = 321)	3.5(1.77,4.22)	3.24(2.49,3.89)	0.02
PLR (*n* = 316)	131.37 (107.32,162.82)	122.03 (98.64,161.48)	0.11
NLR (*n* = 316)	1.73 (1.38,2.24)	1.66 (1.39,2.16)	0.24
SII (*n* = 316)	437.37 (342.65,598.64)	381.38 (296.64,526.21)	0.02

### Differences between invasion and non-invasion groups in pathological parameters

For pathological parameters of DCIS including ER, PR, HER2, DCIS histological grade, and the mixture of histological grades, no statistically significant differences were noticed. Overall, the proportion of patients with positive hormone receptors was much higher than those with negative hormone receptors (68.83% vs. 31.17% for ER and 71.60% vs. 28.40% for PR). As for HER2, patients with a high expression on protein levels (3+) accounted for 46.67% of the whole, well above the theoretical average (25%). Both situations were in line with previous reports (Wolff et al., 2007; Chaudhuri et al., 1993; Wärnberg et al., 2001). Constrained by the cramped space and limited energy supply within the ductal system, the proliferative capacity of DCIS cells was weak, as demonstrated by the low Ki-67 expression in 83.95% of all patients.

### Correlation of hematological indicators with invasion

**Figure 2:**
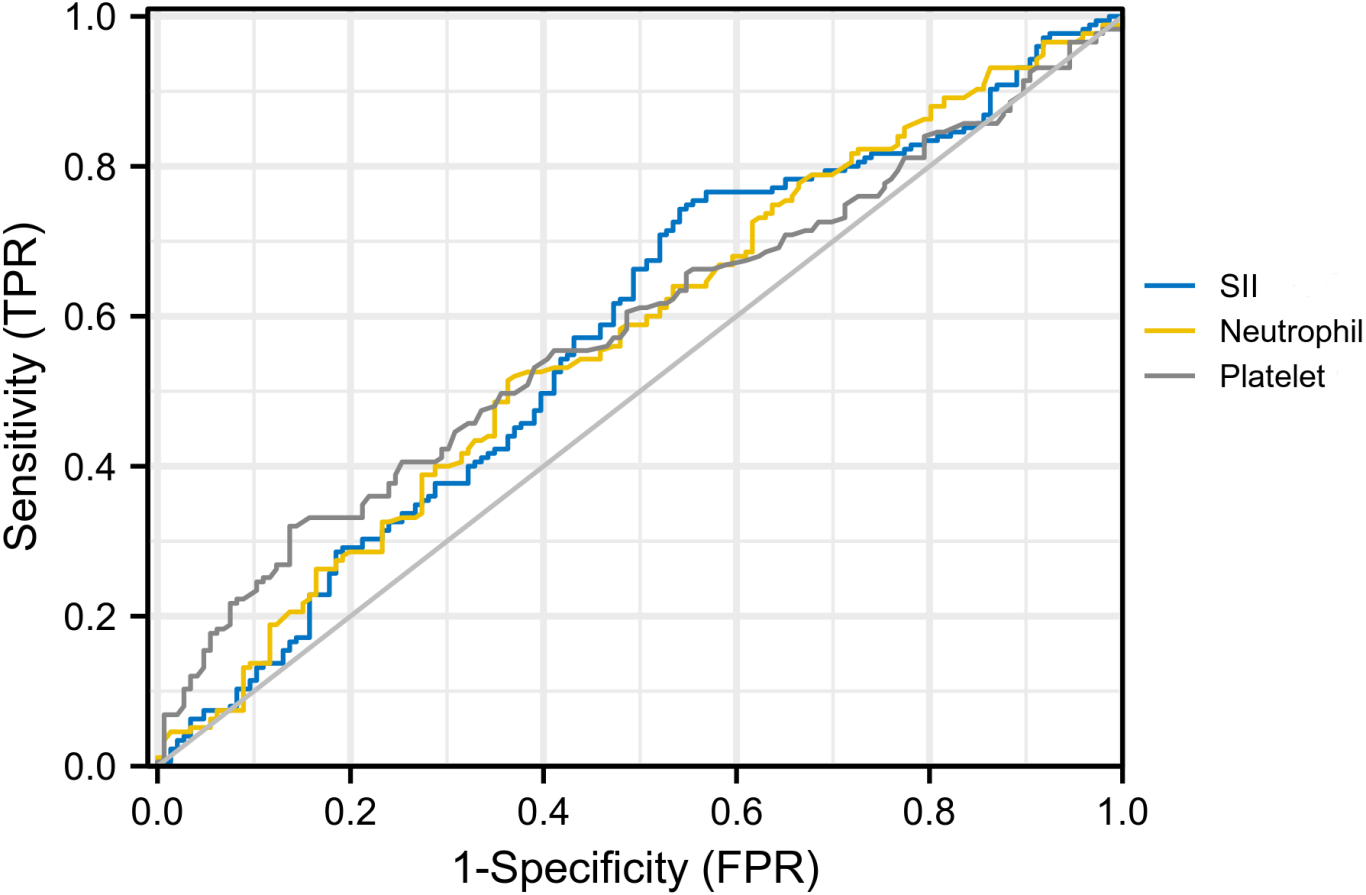
ROC curve for platelet, neutrophil and SII.AUC for SII, platelet and neutrophil were 0.577, 0.567 and 0.579, respectively. Diagonal segments are produced by ties.

For hematological indicators, statistically significant increases in platelet (*p* = 0.03), neutrophil (*p* = 0.02), and SII (*p* = 0.02) in the invasion group were noticed. The receiver operating characteristics curve (ROC) was then used to evaluate the discriminatory power of them and determine the cut-off value with the results shown in Fig. 2. The AUC for platelet, neutrophil, and SII were 0.567, 0.579, and 0.577, with cut-off values of 291.50, 3.49, and 347.20 respectively (corresponding to the maximum of the Youden index). After univariate and multivariate logistic regression analyses, high expression of platelet (>291.50) (odds ratio, 2.46; CI [1.35–4.46]; *p* = 0.003) and SII (>347.20) (odds ratio, 2.54; CI [1.56–4.12]; *p* < 0.001) were established as independent predictors for invasion (Table 2). On this basis, we speculated that invasion of tumor cells into mesenchyme might trigger changes in the body’s immune and inflammatory response.

**Table 2:** Logistic regression for SII, platelet and neutrophil.

	Univariate analysis		Multivariate analysis	
	OR (95%CI)	*p*-value	OR (95%CI)	*p*-value
Platelet (>291.50 vs <291.50)	2.66 (1.51,4.66)	<0.001	2.46 (1.35,4.46)	0.003
Neutrophil (>3.49 vs <3.49)	1.56 (1.00,2.43)	0.049	1.30 (0.81,2.09)	0.286
SII (>347.20 vs <347.20)	2.45 (1.53,3.92)	<0.001	2.54 (1.56,4.12)	<0.001

### The relationship between DCIS and IDC components

To explore changes in the tumor heterogeneity during the transition from DCIS to IDC, we compared immunohistochemical markers between DCIS and IDC components in the invasion group with results shown in Table 3. For ER and PR, similar positive rates in both components were noticed (72.39% vs. 74.63% for ER, *p* = 0.63 and 70.34% vs. 71.19% for PR, *p* = 1.00). The IDC component had a significantly higher expression of Ki-67 compared with the DCIS component (*p* < 0.001).

**Table 3:** Characteristics between DCIS and IDC components in the invasion group.

Characteristics	DCIS (*n* = 177)	IDC (*n* = 177)	*p*-value
ER(*n* = 134)			
Positive	97	100	
Negative	37	34	0.63
PR (*n* = 118)			
Positive	83	84	
Negative	35	34	1.00
Ki-67 (*n* = 130)			
Low	112	91	
High	18	39	<0.01

**Figure 3:**
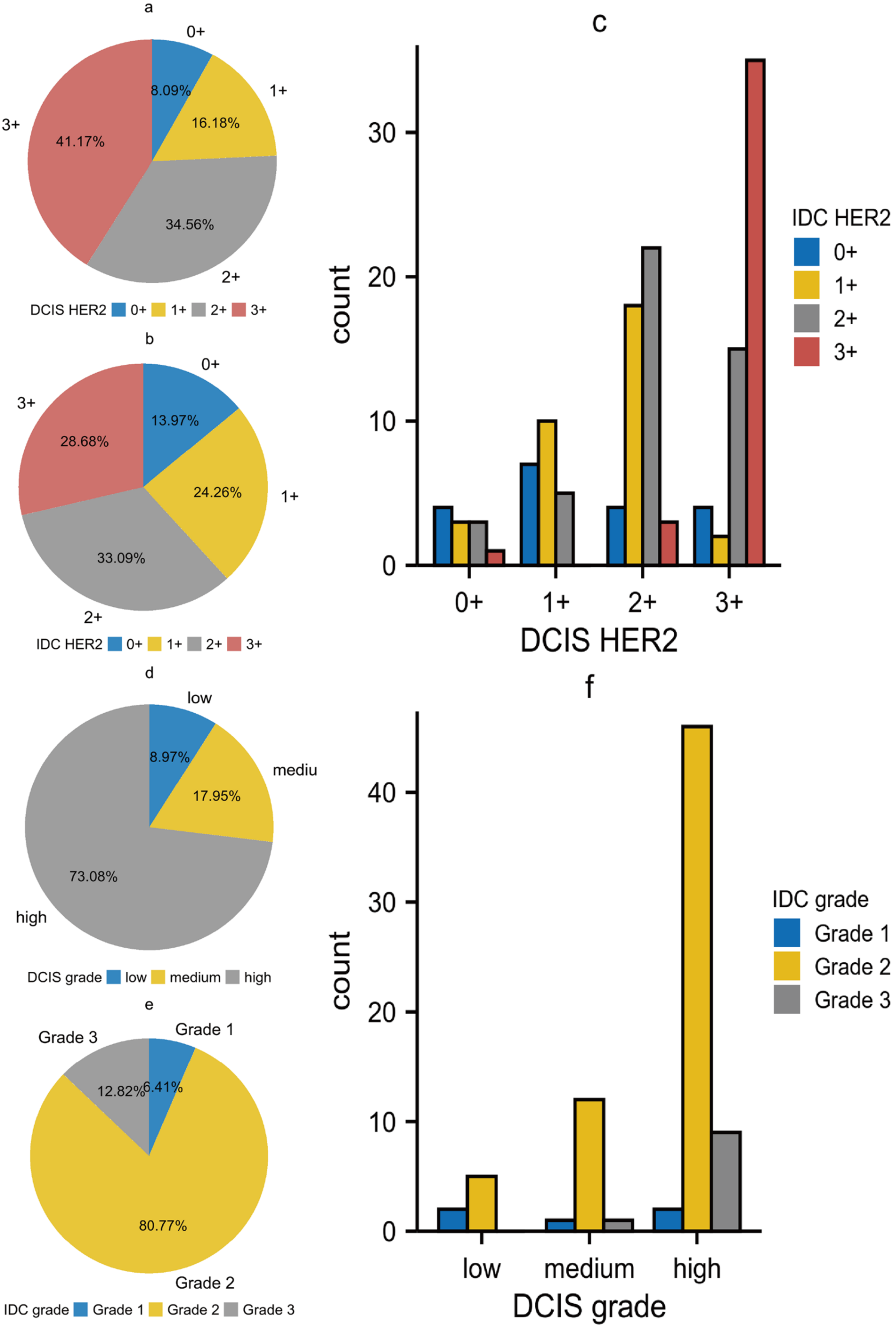
(A–F) The relationship between DCIS and IDC regarding HER2 expression and histological grade.

**Figure 4:**
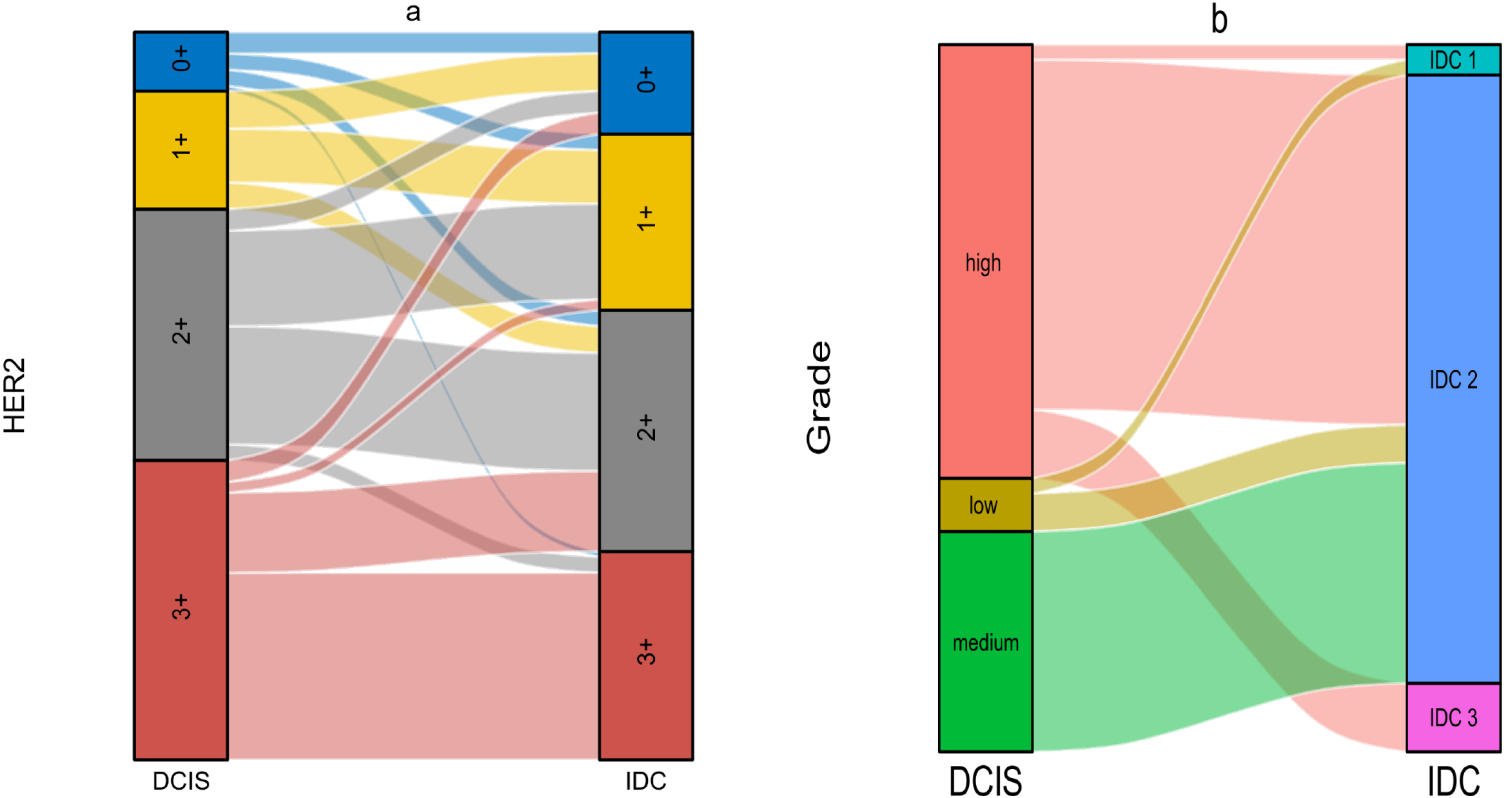
(A–B) The Sankey diagram of relationship between DCIS and IDC.

The relationship between DCIS and IDC regarding HER2 was shown in Table 4. We first performed statistical analysis using Wilcoxon’s test. The result showed *p* < 0.01, indicating that DCIS with different HER2 expression levels would develop into IDC with different HER2 expression levels. The results of Kendall’s tau-U rank correlation and linear by linear chi-square test suggested a positive correlation in HER2 expression between DCIS and IDC components. This correlation was not limited to the level of ’rank’ but was a linear relationship, implying that DCIS with higher HER2 expression tended to develop into IDC with higher HER2 expression. Subsequently, we noted that in the DCIS component, the groups with HER2 expression of 3+ and 2+ accounted for 41.17% and 34.56% respectively. However, in the IDC component, these percentages decreased to 28.68% and 33.09% respectively. In contrast, the groups with HER2 expression of 0+ and 1+ were differentially elevated, which was contrary to our statistical results (Figs. 3A–3C). In the statistical analysis for histological grade, we came to a similar conclusion as for HER2 (Table 5). Although the theory “DCIS with higher histological grades tend to develop into IDC with higher histological grades” holds true, we noticed that the majority of cells in all of the three grades of DCIS tended to develop into IDC grade 2 (Figs. 3D–3F). As a result, in DCIS and IDC components, the high-grade group and IDC grade 2 occupied the highest proportions respectively. We also made Sankey diagrams to show the relationship between DCIS and IDC (Fig. 4). Considering the change in hematological indicators in the invasion group, we speculated the immune and inflammatory response might be responsible for this phenomenon.

**Table 4:** Correlation between DCIS and IDC in the expression of HER2.

		IDC HER2					*p*-value	*p*-value	*p*-value	
		0+	1+	2+	3+	Total	Wilcoxon	Kendall	Chi-square	Kendall’s index
DCIS HER2	0+	4	3	3	1	11				
	1+	7	10	5	0	22				
	2+	4	18	22	3	47				
	3+	4	2	15	35	56				
	Total	19	33	45	39	136	<0.01	<0.01	<0.01	0.54

**Table 5:** Correlation between DCIS and IDC in histological grade.

		Grade of IDC				*p*-value	*p*-value	*p*-value	
		Grade I	Grade II	Grade III	Total	Wilcoxon	Kendall	Chi-square	Kendall’s index
Grade of DCIS	Low	2	5	0	7			
	Medium	1	12	1	14				
	High	2	46	9	57				
	Total	5	63	10	78	<0.01	0.03	0.02	0.23

### Verifications of the predictive power of SII and platelet

To verify the predictive power of hematological indicators, medical records of additional 65 patients were collected. Based on the expression of SII and platelet, they were divided into three groups: Group 1 (SII > 347.2 and platelet > 291.5), Group 3 (SII < 347.2 and platelet < 291.5), Group 2 (the rest of patients). After statistical analysis, the percentage of patients with invasion components was 75% in Group 1, which was significantly higher than 51.85% in Group 2 and 22.72% in Group 3 (*p* = 0.005) (Table 6).

**Table 6:** An independent cohort to verify the predictive power of SII and platelet.

	Group 1	Group 2	Group 3	*p*-value
Invasion	12	14	5	
Non-invasion	4	13	17	0.005

## Discussion

It is of great interest to study the invasion process of DCIS cells from a clinical perspective. In this study, we performed separate comparisons at horizontal and vertical levels to deepen our understanding of this issue. In terms of clinical characteristics, patients were well balanced in baseline characteristics, including age, menstruation, and anatomical subdivisions.

Differences in hematological indicators between the invasion and non-invasion groups were noticed. The expression of platelet and SII was demonstrated to be significantly higher in the invasion group than in the non-invasion group with statistical significance (255 vs. 237 for platelet, *p* = 0.03 and 437.37 vs. 381.38 for SII, *p* = 0.02). This finding was confirmed in the validation cohort subsequently, although without satisfactory AUC values. SII has been recognized as an accessible and independent prognostic factor in a variety of cancers (Gao et al., 2018; Wang et al., 2017; Hu et al., 2014; Hua et al., 2020). It reflects the immune and inflammatory status of the host, which is a well-recognized hallmark of cancer with the ability to influence the formation and progression of tumors at the molecular level, by outlining changes in platelets, neutrophils, and lymphocytes in the circulatory system (Hanahan & Weinberg, 2011; Madhavan & Nagarajan, 2020; Hu et al., 2022; Xie et al., 2022). The pathophysiological roles of platelets, neutrophils and lymphocytes in tumor ecology could improve our understand of SII. Cytokines released by proliferating tumor cells activate platelets, which in turn release VEGF and PDGF to promote angiogenesis in the local lesion and indirectly promote mesenchymal-like transition via the NF- *κ*B and TGF- *β*/Smad pathways (Stanger & Kahn, 2013; Shikada et al., 2005). Neutrophils contribute to the evasion of tumor cells from immune surveillance and promote their invasion and metastasis (Mantovani et al., 2011). Lymphocytes suppress the growth and metastasis of tumor cells by secreting cytokines or cytotoxic effects (Ferrone & Dranoff, 2010). We speculated that the invasion of tumor cells into mesenchyme triggered changes in the immune and inflammatory status of the host, and it was the action of the latter that leaded to concordance in tumor heterogeneity during invasion.

Expression of ER and/or PR in breast cancer is considered a signature of both high differentiation and low proliferation capacities of tumor cells and implies the potential beneficiary from endocrine therapy. The expression rate of ER was reported to be even higher in DCIS than in IDC, reaching 50%–75% (Wiechmann & Kuerer, 2008). In our study, high expression rates of ER and PR in DCIS were noticed (68.86% for ER and 71.60% for PR). In the invasion group, matched DCIS and IDC components expressed both ER and PR at a similar level. However, in the study by Yu et al. (2011) expression of ER was much lower in microinvasion and invasion components compared with in DCIS. Based on this, they speculated that ER-negative cancer cells played a more critical role in the early invasion, which conflicted with our findings. However, both studies were based on a single center with limited sample size. More studies from both clinical and biological perspectives are needed to clarify this puzzle.

HER2 is overexpressed in about 25%–30% of invasive breast cancers with anti-HER2 therapy being proved a promising treatment (Wolff et al., 2007; Krishnamurti & Silverman, 2014). It was reported that the incidence of HER2 overexpression in DCIS reached 60%, which was much higher than in IDC (Latta et al., 2002). However, targeting HER2 was not considered a standard treatment for DCIS yet despite the high expression rate, because we have not yet elucidated the impact of HER2 expression on DCIS cell invasion and long-term prognosis of patients (Siziopikou et al., 2013; Cobleigh et al., 2021; Van Bockstal, Libbrecht & Galant, 2021). In this study, the analysis for the invasion group revealed that although the trend of HER2 expression in DCIS and corresponding IDC was consistent, the proportion of tumor cells with higher expression levels decreased during invasion, seemingly indicating that the altered expression of HER2 was not an “invasion switch” but a result of “external factors”.

Two hypotheses were proposed to help us to understand the invasion process of DCIS. The first one suggested that DCIS cells needed to undergo a stepwise evolutionary pattern from low grade to high grade and finally developed into invasion carcinoma, while the other one suggested that DCIS cells of different histological grades would undergo further genetic mutations and develop into differently differentiated invasive carcinomas respectively (Gupta et al., 1997; Wiechmann & Kuerer, 2008). In our study, the histological grade of DCIS corresponded to the histological grades of IDC respectively, which seemed to support the second hypothesis. Similar to HER2 expression, although a linear relationship between DCIS and IDC in histological grades was demonstrated, the statistical results were not consistent with logical reasoning. The immune-inflammatory response has been shown to be one of the important factors influencing the biological behavior of cells in previous studies (Latta et al., 2002). Considering changes in SII and platelet, we speculated it was the immune and inflammatory response that should be responsible for the phenomenon of HER2 expression and histological grades.

Ki-67 is a proliferation antigen closely related to cell mitosis (Juríková et al., 2016). In clinical settings, it is used to assess the proliferation activity of tumor cells. In our study, the expression of Ki-67 was not significantly different between invasion and non-invasion groups. While the analysis for the invasion group revealed that IDC expressed higher Ki-67 compared with matched DCIS. Two hypotheses were proposed to explain this phenomenon: (a) Tumor cells with stronger proliferative capacity were more aggressive; (b) the increased proliferative capacity was only a consequence of invasion. Tumor cells entering the mesenchyme exhibited enhanced proliferation activity because they were no longer restricted by the cramped space and scarce nutrients within the duct. It has been shown that the limited nutrients in mammary ducts will force tumor cells to choose between “growth or go” in the study by Zanotelli, Zhang & Reinhart-King (2021) which seems to support the second hypothesis.

There are some limitations of this study. First, our study was based on a single center with limited sample size. Second, this study was observational, comparing differences between concurrent DCIS and IDC components without continuous monitoring. Third, we were not able to observe prognostic differences in these patients due to the lack of follow-up data. Fourth, although with statistical significance, the AUC values of hematological indicators were not satisfactory. More multicenter, large-scale clinical trials and biological assays are needed to validate our findings.

## Conclusion

The change in hematological indicators serves as an independent predictor for invasion and can be incorporated into the treatment decision-making process for DCIS. The analysis for Ki-67 reveals that invasion tumor cells exhibit a stronger proliferation capacity compared with the *in-situ* ones. There is no significant difference in the expression of ER and PR but obvious relationships in HER2 expression and histological grades between DCIS and corresponding IDC components. Our study demonstrates that DCIS subclones with different histological grades will develop into invasive carcinomas separately, rather than following a stepwise pattern.

##  Supplemental Information

10.7717/peerj.13966/supp-1 Supplemental Information 1 Raw data for the first comparison

10.7717/peerj.13966/supp-2 Supplemental Information 2 Raw data for the second comparison

10.7717/peerj.13966/supp-3 Supplemental Information 3 Raw data for the validation cohort

## Abbreviations

DCIS: ductal carcinoma *in situ*
IDC: invasive ductal carcinoma
IBTR: in-breast tumor recurrence
SII: systemic inflammatory index
PLR: platelet-lymphocyte ratio
NLR: neutrophil-lymphocyte ratio
ROC: receiver operating characteristic curve
BCS: breast-conserving surgery
ER: estrogen receptor
PR: progesterone receptor
HER2: human epidermal growth factor receptor 2

## Additional Information and Declarations

### Data Availability

The following information was supplied regarding data availability: The raw data are available in the Supplementary Files.
